# Performance of ChatGPT-3.5 and ChatGPT-4 in Solving Questions Based on Core Concepts in Cardiovascular Physiology

**DOI:** 10.7759/cureus.83552

**Published:** 2025-05-06

**Authors:** Arijita Banerjee, Mandrita Chatterjee, Kavita Goyal, Pradosh Kumar Sarangi

**Affiliations:** 1 Physiology, Indian Institute of Technology, Kharagpur, Kharagpur, IND; 2 Physiology, Shri Atal Bihari Vajpayee Government Medical College, Chhainsa, Faridabad, IND; 3 Radiodiagnosis, All India Institute of Medical Sciences, Deoghar, Deoghar, IND

**Keywords:** cardiovascular physiology, core concepts, generative artificial intelligence, large language model, medical education, open-ai

## Abstract

Background

Medical students often struggle to apply previously learned concepts to new situations, such as cardiovascular physiology. ChatGPT, an AI chatbot trained through deep learning, can analyze basic problems and produce human-like language in various subjects. Multiple-choice questions (MCQs) are given to students by many medical schools before exams, but due to time constraints, instructors frequently lack the resources necessary to adequately explain the practice questions. Even when given, the explanations might not give students sufficient information to grasp the concepts completely. This study aimed to examine ChatGPT's ability to solve various reasoning problems based on the core concepts of cardiovascular physiology.

Materials and methods

Multiple-choice questions were presented manually to both chatbots (ChatGPT-4 and ChatGPT-3.5), and the answers generated were compared with the faculty-led answer key using various statistical tests.

Results

The accuracy rates of ChatGPT-4 and ChatGPT-3.5 were 83.33% and 60%, respectively, which were statistically significant. Compared to ChatGPT-3.5, ChatGPT-4's explanation of the response was substantially more appropriate. The execution of ChatGPT-4 was better than ChatGPT-3.5 in certain core concept areas like mass balance (75% vs. 50%), scientific reasoning (60% vs. 40%), and homeostasis (100% vs. 66.67%).

Conclusion

When it came to responding to concept-based questions about cardiovascular physiology, ChatGPT-4 outperformed ChatGPT-3.5. However, to ensure accuracy, faculty members should review the generated explanations, and thus, the growing application of generative AI in the form of virtual-assisted learning approaches in medical education needs to be carefully considered.

## Introduction

Large language model (LLM) generative artificial intelligence (AI) chatbot Chat Generative Pretrained Transformer (ChatGPT) was trained through deep learning to analyze basic problems and produce human-like language in a wide range of subject areas. GPT is a machine learning-trained system that can use its cognitive abilities to answer any question, provide prompt answers, and aid in virtual teaching. The OpenAI chatbot, ChatGPT, was first released in 2022. The application is free to use, which has actually made it more widely accepted worldwide. This AI application could revolutionize publishing, value-added learning, and research in the higher education system [1,2].

Medical students frequently struggle to apply previously learnt concepts to new situations. For instance, students who study the fundamentals of hemodynamics in cardiovascular physiology find it difficult to apply those same concepts to the pulmonary system's gas exchange and airflow. Though there is much to learn about cardiovascular physiology, a few basic concepts are useful for comprehending hemodynamics in a clinical setting. According to Michael and Farland, conceptual learning from one physiological system to another can be enhanced by concentrating on the fundamental ideas [3]. In the field of physiology, where knowledge is extensive and constantly growing, the innate ability to comprehend and recognize that something previously learnt can later be applied to something new is a valuable and potent skill. Homeostasis, cell membrane, mass balance, flow down gradients, and cell-to-cell communication are the five fundamental concepts that Michael and a number of other people have developed and applied in various modules of physiology [4]. A fundamental knowledge of cardiovascular physiology is essential for managing patients during the perioperative period. The cardiovascular system's main function is to supply enough blood flow to meet the metabolic demands of the body's tissues. Several governing principles are applied in clinical scenarios based on cardiac electrophysiology, the major determinants of stroke volume, indices of cardiac function, ejection fraction, major circulatory reflexes, role of capillaries in fluid absorption, and myocardial contractility as measured by cardiac function curves [5].

In contrast to memorization, competency-based medical education (CBME) in India emphasizes the development of specific competencies that call for a conceptual grasp of a variety of subjects. Most ChatGPT studies have used this design for multiple-choice, standardized questions [6]. Multiple-choice questions (MCQs) are given to students by many medical schools before exams, but due to time constraints, instructors frequently lack the resources necessary to adequately explain the practice questions. Even when given, the explanations might not give students sufficient information to grasp the concepts completely. We attempted to investigate in this study how well both old and new versions of ChatGPT solve different reasoning problems based on the fundamental ideas of cardiovascular physiology. Additional fundamental ideas are emphasized to assist students and teachers in creating efficient teaching resources in physiology for the transfer of knowledge.

## Materials and methods

Study design

The cross-sectional observational study was carried out in July 2024 at the Dr. B. C. Roy Multi-speciality Medical Research Centre, Indian Institute of Technology (IIT), Kharagpur, in the Department of Physiology.

Ethical consideration

Since this study did not use any human or animal research subjects, ethical approval was not required.

Study tools

Our study used both the paid version of ChatGPT (ChatGPT-4) and the free version (ChatGPT-3.5) from OpenAI. A set of single-choice questions was given to two iterations of OpenAI's ChatGPT, and their answers were assessed and compared.

Preparation of questions based on core concepts in cardiovascular physiology

The multiple-choice question (MCQ) bank was created through a methodical process that the research team carried out. The group used a range of reputable textbooks to find questions. These sources included the well-known "Berne and Levy Textbook of Physiology," "Guyton and Hall Textbook of Medical Physiology," and "Ganong Medical Physiology." Homeostasis, flow down gradients, cell membrane, mass balance, energy, scientific reasoning, system integration, and cell-to-cell communication in cardiovascular physiology are the eight fundamental concepts linked to the 30 MCQs in English that the faculty of the department of physiology selected, as illustrated in Table 1.

**Table 1:** Core concepts in cardiovascular physiology

Homeostasis	Flow down gradients	Cell membrane	Cell-to-cell communication	Mass balance	Energy	Scientific reasoning	System integration
Body water composition	Basic flow equation	Transport across the membrane	Gap junctions	Action potential	Exercise and cardiovascular response	Baroreceptor reflex	Hypertension
Diffusion	Poiseuille equation	Voltage-gated channels	Cardiac cell	Electrocardiography	Gravity and cardiovascular response	Cardiac output regulation	Circulatory shock
Negative feedback regulation	Fick principle	Ligand-gated channels	Calcium-induced calcium release	Control of cardiac output	Malignant hyperthermia	Blood pressure regulation	Cardiac autonomic neuropathy

The faculty of the Department of Physiology prepared the answer keys to all of the questions on ChatGPT prior to the test starting. To guarantee correctness and relevance to the subject, every MCQ and its accompanying answer key were carefully examined. The LLMs used in this study did not have image recognition capabilities. Consequently, questions that included tables or figures were not included. Each LLM's accuracy rate served as the main outcome measure.

Data collection

The questions were presented manually to both chatbots on two specific days of the week, and the first response provided by each tool was considered final; the feature to "regenerate response" was not used; if the results obtained matched the faculty's previously provided answers, it was taken to be correct. In addition, all of the explanations provided by both LLMs were checked separately by the subject experts; thus, the number of correct responses, incorrect responses, and appropriate and inappropriate explanations, among others, was calculated. Each question had a choice of four possible answers, of which one was correct. For example, with reference to the core concept of mass balance, one of the questions was asked as follows: A 50-year-old man's ECG was obtained with conventional bipolar limb leads, and it was discovered that the three standard leads' combined voltage was 4 millivolts. Four possible answers given were a) right ventricular enlargement, b) normal heart, c) right atrial hypertrophy, and d) left ventricular hypertrophy. In this case, option b was the correct answer.

Statistical analysis

Frequency distributions and percentages were used to characterize categorical variables, and the distributions of each group were compared using the chi-square or Fisher's exact test. IBM SPSS Statistics for Windows, version 28.0 (released 2021, IBM Corp., Armonk, NY) was used to analyze the data. P-values below 0.05 were regarded as statistically significant.

## Results

In Table 2, the comparison of the overall correct answers for each LLM is depicted. The accuracy rates of ChatGPT-4 and ChatGPT-3.5 were 83.33% and 60%, respectively. There was a statistically significant difference in the accuracy rates between ChatGPT-4 and ChatGPT-3.5 (P = 0.045). Both LLMs could give 75% and 100% correct responses to questions in homeostasis, flow-down gradient, cell membrane, and energy, respectively. The execution of ChatGPT-4 was better than ChatGPT-3.5 in certain core concept areas like mass balance (75% vs. 50%), scientific reasoning (60% vs. 20%), system integration (75% vs. 25%), and homeostasis (100% vs. 66.67%).

**Table 2:** Comparison of correct responses between ChatGPT-4 and ChatGPT-3.5 *p < 0.05 is significant.

Core concepts	ChatGPT-4 N (%)	ChatGPT-3.5 N (%)	Chi-square value	p-value
Total (N = 30)	25 (83.33)	18 (60)	4.022	0.045^*^
Homeostasis (N = 3)	3 (100)	2 (66.67)		
Flow-down gradient (N = 4)	3 (75)	3 (75)		
Mass balance (N = 4)	3 (75)	2 (50)		
Energy (N = 3)	3 (100)	3 (100)		
Cell membrane (N = 3)	3 (100)	3 (100)		
Cell-to-cell communication (N = 4)	4 (100)	3 (75)		
Scientific reasoning (N = 5)	3 (60)	1 (20)		
System integration (N = 4)	3 (75)	1 (25)		

Compared to ChatGPT-3.5, ChatGPT-4's explanation of the response was substantially more appropriate, as shown in Table 3. ChatGPT-4 could give the explanations appropriately to 23 questions, while ChatGPT-3.5 gave correct explanations to only 16 questions, irrespective of the accuracy of answers. Of all the questions, in the case of ChatGPT-4, 76.67% of the explanations covered every facet of the faculty-generated response, while 13.33% of the explanations were correct and covered at least some of the faculty-generated response. The statistical difference was not quite bit significant, though.

**Table 3:** Comparison between ChatGPT-4 and ChatGPT-3.5 regarding the adequateness of the explanations

Total questions (n)	ChatGPT-4 (n/%)	ChatGPT-3.5 (n/%)	Chi-square value	p-value
30	23 (76.67)	16 (53.33)	3.60	0.05

The explanations and answers were both correct and adequate for 21 questions in the case of ChatGPT-4 as compared to 15 questions for ChatGPT-3.5. Four questions for ChatGPT-4 while three for ChatGPT-3.5 had the right answers, but the explanations were not appropriate. Out of 30 questions, ChatGPT-4 gave both incorrect answers and explanations to only three questions in comparison to ChatGPT-3.5, which gave both wrong answers and explanations to 11 questions, as illustrated in Table 4.

**Table 4:** Comparison of responses and adequateness of explanations n = number of answers to the questions, % = percentage

Adequateness of explanations	ChatGPT-4 (n/%)	ChatGPT-3.5 (n/%)
Correct answer and appropriate explanation	21 (70)	15 (50)
Correct answer and inappropriate explanation	4 (13.33)	3 (10.00)
Incorrect answer and appropriate explanation	2 (6.67)	1(3.33)
Incorrect answer and inappropriate explanation	3 (10.00)	11 (36.67)

## Discussion

The performance of ChatGPT-3.5 and ChatGPT-4 in answering questions pertaining to fundamental ideas in cardiovascular physiology is being assessed and compared for the first time in this study. In this study, ChatGPT-4 outperformed ChatGPT-3.5. ChatGPT-4 and ChatGPT-3.5 had overall performance rates of 83.33% and 60%, respectively. These scores are significantly higher than the 50% needed to pass any competitive medical exam. In line with our results, Toyama et al. found that the performance of ChatGPT-4 was better than ChatGPT-3.5 and Bard (65.0%, 40.8%, and 38.8%, respectively) with respect to radiology examination [7].

Regardless of the language, ChatGPT-4 surpassed ChatGPT-3.5 in both the Polish and English versions of the Specialty Certificate Dermatology Examination. ChatGPT-4 outperformed ChatGPT-3.5 in a study that used an American College of Emergency Medicine question bank (82.1% vs. 61.4%). Bing Chat has been included in a small number of earlier studies. The authors decided not to include Bing Chat in the current study because both ChatGPT-4 and Bing Chat use the same language learning model [8,9]. However, Bing Chat performed better than Bard and ChatGPT-3.5 in the English portion of the Royal College of Ophthalmologists fellowship exam, according to Raimondi et al. The reason for this discrepancy in responses could be that different businesses use these two LLMs, and each chatbot's interface, approach to user interaction, data collection, and processing approach for enhancement may vary [10].

Banerjee et al. found that ChatGPT-3.5 could respond to 77% of the conceptual questions pertaining to the fundamental ideas of entire medical physiology, which is in accordance with the present study (76.67% for ChatGPT-4, 53.33% for ChatGPT-3.5). According to earlier research, the core concepts (60 questions) had an overall mean score of 3.68 ±0.30, which was below 4.00 and called for additional machine learning-based ChatGPT training. Cardiovascular physiology had the highest scores out of the six modules, followed by neurophysiology. "Mass balance" received the lowest score among the fundamental concepts, while "cell-to-cell communications" and "cell membrane" received the highest scores. In line with the findings of the previous study, both LLMs were able to provide 75% and 100% accurate answers to questions about flow-down gradients, cell membranes, and cell-to-cell communication, respectively, in the current study. When it came to some fundamental concepts, such as mass balance (75% vs. 50%) and homeostasis (100% vs 66.67%), ChatGPT-4 outperformed ChatGPT-3.5. It did particularly well in modules that focused on physiology core concepts, like homeostasis and flow-down gradients, which are highly sought after for answer explanations because they require multi-step reasoning that can take a long time to complete. The notable disparities in fundamental ideas underscore the need for additional assessment of AI-enabled learning applications by highlighting the disparate ways in which the same software tool is executed and the overly detailed responses [11].

Compared to ChatGPT-3.5, ChatGPT-4 had a substantially more appropriate explanation for the response. This result is in line with a recent study that found ChatGPT-4 performed better than ChatGPT-3.5 on higher-order radiologic questions. Correct answers were either accompanied by appropriate explanations or inappropriate, while incorrect answers were occasionally associated with adequate explanations. When it comes to correctly answered questions, ChatGPT-4's explanations are at least as good as those produced by faculty in 70% (21 questions) of instances compared to 50% in the case of ChatGPT-3.5, thus indicating its potential as a supportive tool in medical education [12,13].

Artificial hallucination, which can be especially prevalent in generative models like LLMs, happens when chatbots produce incorrect information with facts as if it were true. It can show up in a number of scenarios, including when LLMs are performing complex tasks, when the training environment differs from the real work environment, or when they are not trained with enough or accurate data. According to a study by Krisha Mohan et al., ChatGPT received a distinction (>75% of marks) on the Physiology University Examination. Mahat et al. assessed ChatGPT's performance in answering phase I Bachelor of Medicine and Bachelor of Surgery (MBBS) biochemistry exam questions and found that it passed the Biochemistry University Exam with an average score [14-16].

The field of AI is developing quickly, and ChatGPT's LLM has recently undergone a number of upgrades. More accuracy, a deeper comprehension of context, and more cohesive responses in intricate conversations are just a few of the notable enhancements that ChatGPT-4 offers over ChatGPT-3.5. It is more effective, responds more quickly, and has superior reasoning and problem-solving skills. Because of these developments, ChatGPT-4 is especially attractive for more complex applications (like technical writing and customer support, for example). However, ChatGPT-4 requires a subscription from the user. An improved version of GPT-4o, where "o" stands for "omni," can generate any combination of text, audio, image, and video as output and accept any combination of these as input [17,18].

There are certain limitations to the study. First, it only had a few questions, and each LLM only had one answer to the questions. This might not accurately represent the LLMs' capacity to address medical issues. The performance of LLMs is getting better every day, so if the study is done again soon with the same methodology, different results might be found. Second, we did not include questions with images, like radiographs, CT scans, ultrasounds, or electrocardiograms, which are frequently used in medical education. It is also necessary to evaluate other LLMs, such as Bard, Bing, Gemini, LaMDA, and Claude. Since professor-generated practice questions are regarded as intellectual property of the university, we advise faculty to disable their chat history when using this tool to avoid content being used in subsequent model training.

## Conclusions

Understanding that students will transfer what they have learnt is crucial for facilitating the transfer of physiology learning. Fundamental ideas serve as the foundation for this knowledge, which requires greater accuracy and precision when utilizing AI-enabled learning tools. When it came to responding to concept-based questions about cardiovascular physiology, ChatGPT-4 outperformed ChatGPT-3.5. Yet, ChatGPT-4 is meant to be a tool to support faculty and possibly cut down on the amount of time required to create explanations for practice questions, not to replace them. Even though LLM performance is getting better every day, they continue to generate inaccurate results, so their growing application in virtual assisted teaching, clinical practice and education needs to be carefully considered.
